# The Association Between Parental Generativity and Parent-Child Attitude-Similarity Through Parent- and Child-Reported Authoritative Parenting: A Replication

**DOI:** 10.5964/ejop.8375

**Published:** 2023-11-30

**Authors:** Holger Busch

**Affiliations:** 1 Department of Psychology, Trier University, Trier, Germany; Università Cattolica del Sacro Cuore, Milan, Italy

**Keywords:** generativity, parenting style, authoritative parenting, parent-child similarity, serial mediation, replication

## Abstract

Generativity is the desire to pass something on to the coming generations. Through parents’ and children’s reports on authoritative parenting, parents’ generativity is associated with how similar young adults think their attitudes are to those of their parent (Peterson et al., 1997; https://doi.org/10.1037/0022-3514.72.5.1202). The present study represents a direct replication of these results. Altogether, a sample of 365 German parent–child dyads participated in the study (parents’ age: M = 52.87, SD = 4.89; children’s age: M = 20.81, SD = 2.26). Parents provided information on their generativity (Loyola Generativity Scale) and parenting styles (Parental Authority Questionnaire). Their child provided information on perceived parenting styles (Parental Authority Questionnaire) and attitudinal similarity to the parent (Psychological Separation Inventory). A serial mediation was found for authoritative parenting. It was not found, however, for authoritarian and permissive parenting. This pattern replicates Peterson et al.’s (1997) results. Potential questions for future research on how generative adults transmit their values and attitudes are discussed.

In his theory of psychosocial development, Erikson (1963) distinguishes eight developmental crises each individual has to cope with across the life-course. Middle adulthood is dominated by the crisis of generativity vs. stagnation. That is, adults are concerned with the well-being of younger generations and what they can contribute to younger generations. In other words, generative individuals develop the intention to pass something on to future generations that hopefully these can benefit from. Stagnation, on the other hand, means that people increasingly and eventually exclusively care for themselves.

That said, generative efforts include two parties: One is the generative actor, the other is the intended recipient. Importantly, the term recipient is not meant to imply passivity on the recipient’s side. On the contrary, recent research suggests that the recipient’s personality affects how much an individual benefits from somebody else’s generative efforts (Busch & Hofer, 2022; Thomas et al., 2022).

Of course, generative individuals can address various recipients in their generative efforts such as their children or junior co-workers. Moreover, generative individuals can have more or less specific recipients in mind when behaving generatively (e.g., by giving some piece of advice to their child or by publishing a book to communicate their experiences and what to learn from them to a wide readership). Albeit little is known about how generative actor–recipient dyads form, this might partly depend on the generative individual’s social motives (Peterson & Stewart, 1993).

Most research on generativity focuses exclusively on the generative individual. This line of research has yielded valuable insight into how generativity relates to personality traits and/or well-being of the generative individual (e.g., de St. Aubin & McAdams, 1995). For example, regarding traits, generativity is positively associated with extraversion, openness, and conscientiousness, but negatively associated with neuroticism (Cox et al., 2010). Concerning well-being, generativity is related to various indicators of well-being such as life satisfaction and purpose in life (e.g., Busch & Hofer, 2012; Cox et al., 2010; de St. Aubin & McAdams, 1995).

The recipient of generativity, however, has only rarely been acknowledged in research. This is unfortunate given that the recipient is likely to have an effect on the generative individual. For example, Cheng (2009) investigated how generative individuals’ well-being was affected when they perceived young people, that is, potential recipients of their generative efforts, to be disrespectful. Indeed, when they perceived young people as disrespectful, people’s generative efforts decreased over a one-year-interval. This finding has since been corroborated in another longitudinal study (Tabuchi et al., 2015). In line with these findings, when passing on experiences from a failure episode in their lives to a younger person, individuals’ generativity scores depended on the recipient’s reaction. Specifically, individuals confronted with a neutral recipient reaction showed a decrease in generativity, whereas generativity increased from baseline to post-interaction assessment when the recipient reacted positively (i.e., smiling, nodding) to the generative individual (Tabuchi & Miura, 2016). Taken together, the studies reviewed above highlight the importance of the recipient’s reaction for the effects that their generative efforts have for the generative individual.

However, in these studies, again, the focus has been on the effect that generativity has on generative individuals rather than the effects that generativity has on the recipient. Focusing on the effects that generativity has on the recipient, Kessler and Staudinger (2007) found that adolescents demonstrated more willingness to help someone after a generative conversation with an adult than after a non-generative conversation with an adult or a conversation with a peer. That is, the generative action of the adult had an immediate impact on the adolescent’s subsequent behavior.

Provided that generative interaction between two individuals takes place continuously for a long period of time, one would thus assume that generativity should have a lasting effect on the recipient. For instance, Thomas et al. (2022) found that adolescents who participated in an intergenerational exchange program had a stronger increase in self-concept clarity than control group adolescents. Jones and McAdams (2013) reported that adults scored higher on generativity if they themselves had benefitted from somebody else’s generative efforts. Busch and Kranz (2022) found a similar effect of generativity among gay men: Gay men had a more positive identity and were more generative if they had been the recipient of somebody else’s generativity. Hebblethwaite and Norris’s (2011) interview data suggest that generative interactions with their grandparents inspired generative intentions in young adult grandchildren. A good context to test the assumption of a lasting effect of generativity on the recipient is parenting as it provides the generative adult with the on-going opportunity to help a younger person thrive.

In two studies, Peterson has provided evidence that generative parents indeed tend to be successful in passing on their attitudes to their children: In one study, parents’ generativity was associated with their young adult children’s positive affect across a four-year-interval. Moreover, parents’ generativity predicted their transmission of political values to their children, which in turn predicted children’s interest in politics across the four-year-interval (Peterson, 2006).

In the other study, Peterson et al. (1997) showed that parents’ generativity related to their children’s evaluation of how closely their attitudes on political, moral, or social issues resembled those of their parent. Their data from 120 dyads of a parent and their young adult child also supported their hypothesis that generative adults tended to report an authoritative parenting style (for further evidence, see Pratt et al., 2001). Parents’ authoritative parenting was in turn perceived as such by their children, and this perception was associated with higher children’s ratings of attitudinal similarity with their parent (cf. more recent research on the importance of the perceived parenting style on identity development, Lehmann et al., 2021). That is, Peterson et al. (1997) provided important evidence that generativity can indeed have a lasting impact on the recipient in that children reported to hold attitudes similar to those of their parent, which is mirrored in Peterson’s (2006) findings that parents’ generativity correlated with their evaluation of how similar their child was to them. Moreover, for the context of parenting, Peterson et al. (1997) specified a mechanism how this impact is achieved.

Finally, Peterson et al. (1997) demonstrated that the effect was restricted to authoritative parenting, but did not occur for authoritarian or permissive parenting. According to Baumrind’s (1971) definition, each parenting style is characterized by parental demandingness (i.e., clear maturity standards and monitoring of the child’s behavior) and responsiveness (i.e., the amount of emotional warmth and support they provide the child with). A combination of high demandingness and high responsiveness, that is, authoritative parenting, might be most successful in establishing child-parent similarity because the child may feel free to explore alternative attitudes (unlike with authoritarian parenting: high demandingness and low responsiveness), but still have parental guidelines or suggestions as to which attitude might benefit them (unlike with permissive parenting: low demandingness and high responsiveness).

## Aims of the Present Research

Given that the intention to pass something on to future generations is the core of generativity, it seems a regrettable neglect that so few studies have tested the impact an adult’s generative efforts have on the recipient. Thus, as its findings are so close to the heart of generativity, the present study is based on the conviction that the results of Peterson et al. (1997) deserve a replication attempt (on the importance of replication as a tool to verify empirical results, see, e.g., Schmidt, 2009).

Specifically, the present study is a direct replication (Schmidt, 2009) in that it employs the same operationalizations as the original study. There are, nonetheless, some deviations from the original study that are worth mentioning: The present sample comes from a different cultural background, that is, Germany instead of the USA, providing information on the generality of Peterson et al.’s findings. Finally, Peterson et al. (1997) yielded their results via a series of simple regressions. The present study uses a more informative approach by applying Hayes’s (2022) PROCESS to examine the serial mediation.

For replication attempts, it is important to aim at a higher power than the original study to account for various uncertainties (Anderson & Maxwell, 2017). To determine an adequate sample size, Schoemann et al.’s (2017) tool for mediation analysis power calculations was employed. Peterson et al.’s (1997) original correlation coefficients were used to determine the minimum sample size for a .90 power for the serial mediation. As analyses yielded *N* = 350, this was the aim for the recruitment of participants.

The present research thus is guided by the hypothesis that a serial mediation is found with parents’ generativity as predictor, children’s attitudinal similarity to their parents as outcome, and parents’ and children’s reports on authoritative parenting as two mediators. On the contrary, no such serial mediation is expected for the authoritarian and permissive parenting styles.

It ought to be stressed that the present study does not replicate Peterson et al.’s (1997) findings in its entirety: The original study also assessed political interest, authoritarianism, and children’s evaluations of frequency of conflict with parents. As, however, the mediating link via parenting was not tested for political interest and parental authoritarianism was not related to attitudinal similarity, these variables are not considered in the present study. Finally, as the present study’s focus is on generativity’s contribution to successful intergenerational transmission, the conflict variable is not considered either. Thus, the aim of the present study is to replicate Peterson et al.’s finding that parental generativity is associated with child-parent attitudinal similarity through authoritative parenting as reported by parents and children.

## Method

### Participants

Originally, data were collected from 365 parent–child dyads. Because of missing data, however, the data of 12 dyads could not be used for analyses. One parent and two children failed to provide any sociodemographic information. With respect to parents, 68% of participants were mothers, 32% fathers. Concerning education, most parents (60%) had acquired a university-entrance diploma (Abitur) or a university degree. Parents’ age ranged from 40 through 73 years (*M* = 52.87, *SD* = 4.89).

The young adult children were predominantly female (86%). They were between 18 and 31 years old (*M* = 20.81, *SD* = 2.26). All young adult children were university students; most of them majored in the Humanities (52% psychology, first-term students only; 28% educational sciences; 14% social sciences). Other majors were, for example, business administration, economics, engineering, and physics. Young adult children had up to seven siblings (*M* = 1.39, *SD* = 0.99).

Most parent–child dyads were same-sex dyads (67%). Specifically, there were 214 mother–daughter and 20 father–son dyads as well as 90 father–daughter and 26 mother–son dyads.

### Procedure

The young adult children were recruited at two German universities in 2018. They were given one questionnaire for themselves and one for their parent. They returned their parent’s questionnaire in a closed envelope so they could not see their parent’s responses. Young adult children received partial course credit for their and their parent’s participation.

Participants were informed about the purpose of the study. They provided informed consent prior to their participation. The procedure conformed to the ethical guidelines of the German Psychological Association (2018).

### Measures

#### Generativity

The Loyola Generativity Scale (LGS; McAdams & de St. Aubin, 1992) was applied to assess parents’ disposition for generativity (German translation: Hofer et al., 2008). The LGS consists of 20 items (sample item: “I have made and created things that have had an impact on other people”) which are evaluated on a four-point Likert scale from 0 (never) to 3 (very often/nearly always). High scores indicate a pronounced generative concern. Cronbach’s Alpha was .84.

#### Parenting Styles: Parents’ Perspective

To assess the authoritative, authoritarian, and permissive parenting styles, the Parental Authority Questionnaire (PAQ; Buri, 1991) was employed. As the PAQ is originally phrased from the child’s perspective (see below for sample items), items were rewritten to capture the parent’s first-person perspective. Moreover, parents were instructed to “think back to when your child was still living with you.” The thirty items are rated on a five-point Likert scale from 0 (strongly disagree) to 4 (strongly agree). Each parenting style is covered by ten items. High scores indicate that a given parenting style was highly endorsed by the parent.

Because to the best of the present author’s knowledge, no previous study has adapted the PAQ items to capture parents’ reports on their parenting styles, a principal components analysis was run (Kaiser-Meyer-Olkin criterion: .87; anti-image-correlations ≥ .62) with number of factors set at 3. The first factor represented authoritarian parenting. Its eigenvalue was 5.56, explaining 19% of variance. Factor loadings were ≥ .59 on their designated factor, cross-loading were ≤ |.32|. The second factor represented authoritative parenting with an eigenvalue of 3.89 and 13% of explained variance. Two items were problematic in that cross-loadings with the authoritarian factor were equal to or higher (Item 8: .57; Item 15: .39) than their factor loadings with the designated authoritative parenting factor (Item 8: .30; Item 15: .39). Factor loadings for the remaining 8 items were ≥ .52 on their designated factor with cross-loadings ≤ |.36|. The third factor represented permissive parenting (eigenvalue: 3.13; explained variance: 10%) with factor loadings on their designated factor ≥ .28 and cross-loading ≤ |.28|. Cronbach’s Alphas were .79 (authoritative), .88 (authoritarian), and .70 (permissive), respectively. The serial mediation analysis for authoritative parenting was rerun with a reduced version of scales, in which the two problematic items were removed in both the parent and the child questionnaire. As results did not differ from those with the full scales, the latter are reported below. The former are documented and can be found in the Supplementary Materials. There was missing data on the authoritarian parenting scale for five parent participants, and on the permissive parenting scale for six parent participants.

#### Parenting Styles: Children’s Perspective

To assess the authoritative, authoritarian, and permissive parenting styles from the children’s point of view, the original PAQ (Buri, 1991; German translation: Castello & Hubmann, 2014) was used. Young adult children were also instructed to “think back to when you were still living with your parents.” Moreover, they were instructed to refer only to the parent that also participated in the study. High scores indicate that the child considered a given parenting style highly pronounced in their parent. Cronbach’s Alphas were .79 (authoritative; sample item: “My parent gave me direction for my behavior and activities as I was growing up and he/she expected me to follow his/her direction, but he/she was always willing to listen to my concerns and discuss that direction with me”), .87 (authoritarian; “As I was growing up, I knew what my parent expected of me in the family and he/she insisted that I conform to those expectations simply out of respect for his/her authority”), and .77 (permissive; “My parent feels that most problems in society would be solved if parents would not restrict their children’s activities, decisions, and desires as they are growing up”), respectively. One child participant produced missing data on the authoritarian parenting scale.

#### Attitudinal Similarity

Children’s self-reported attitudinal similarity was measured with the corresponding subscale of the Psychological Separation Inventory (PSI; Hoffman, 1984). Fourteen items concerning, for example, political, moral, and religious attitudes (sample item: “My opinions regarding the role of women are similar to my parent’s”) are rated on a five-point Likert scale ranging from 0 (not at all true of me) to 4 (very true of me). Again, young adult children were instructed to refer to the parent that also participated in the study. High scores indicate that the child views their attitudes to be similar to that of their parent. The items were translated into German via back-translation procedure. A principal components analysis was run (Kaiser-Meyer-Olkin criterion: .89; anti-image-correlations ≥ .80) with number of factors set at 1. The eigenvalue of this factor was 5.56. The factor explained 40% of variance (factor loadings ≥ .52). Cronbach’s Alpha was .88.

## Results

The results section is organized as follows: First, correlations among the psychological variables are given. Then, gender differences are examined. After these preliminary analyses, the serial mediation model is tested for authoritative, authoritarian, and permissive parenting styles, respectively.

Descriptive statistics are given in Table 1, along with the correlations among measures. Concerning sociodemographic variables, parents’ age was correlated with their authoritarian, *r* = –.11, *p* = .041, and permissive parenting, *r* = .14, *p* = .011, but not their generativity or authoritative parenting. Children’s age was not significantly correlated with any scale. Children’s number of siblings was positively correlated only with parents’ generativity, *r* = .15, *p* = .004. For parents, a small (Cohen, 1988) gender difference effect emerged for authoritative parenting, *t*(350) = 3.02, *p* = .003, *d* = 0.346, with mothers (*M* = 2.75, *SD* = 0.52) reporting more authoritative parenting than fathers (*M* = 2.56, *SD* = 0.55), but not for generativity, authoritarian, or permissive parenting. In the child sample, gender differences were found neither for perceived authoritative, authoritarian, or permissive parenting, nor for attitudinal similarity to their parent. To account for the imbalance in the gender distribution, a random subsample of 111 mothers was drawn from the sample. Thus, gender difference tests were rerun with an equal number of mothers and fathers, all with complete data on all scales. These results confirmed the significant gender effect on authoritative parenting, but yielded no additional gender effect. Analogously, a random subsamples of 46 female children was drawn and compared with the same number of male children in the sample. Again, this rerun of analyses yielded no additional gender differences in the child sample. In sum, the associations between sociodemographics and the psychological variables under investigation were generally insignificant or small.

**Table 1:** Descriptive Statistics and Correlations for Parent (Generativity, Parenting Styles) and Child (Parenting Styles, Attitudinal Similarity) Scales

Variable		*M* (*SD*)	1	2	3	4	5	6	7	8
Parent measures										
1	Generativity	1.78 (0.41)	—							
2	Authoritative parenting	2.69 (0.54)	. 33***	—						
3	Authoritarian parenting	1.39 (0.72)	–.01	.15**	—					
4	Permissive parenting	1.83 (0.53)	–.04	–.07	–.15**	—				
Child measures										
5	Authoritative parenting	2.67 (0.59)	.12*	.35***	–.23***	–.03	—			
6	Authoritarian parenting	1.67 (0.72)	–.01	–.04	.49***	–.22***	–.23***	—		
7	Permissive parenting	2.03 (0.57)	.02	.04	–.28***	.30***	.14*	–.50***	—	
8	Attitudinal similarity	2.70 (0.68)	.15**	.24***	–.18**	.01	.57***	–.23***	.23***	—

*Note*. Due to missing data (see methods for details), *N* ranges from 343 through 353 for correlations. Following Cohen’s (1988) conventions, correlation coefficients of .10 are considered small, of .30 moderate, and of .50 large, respectively.

*p < .05. **p < .01. ***p < .001.

The hypothesized serial multiple mediator model was tested using Hayes’ (2022) PROCESS for SPSS which computes two simple mediation indirect effects in addition to the serial two mediators model (Model 6). All indirect effects were tested with 10,000 bootstrap samples. Variables were standardized before running the mediation.

**Figure 1:**
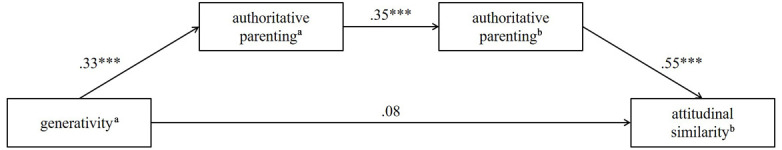
Standardized Regression Coefficients for the Serial Multiple Mediators Model for Authoritative Parenting ^a^As reported by parents. ^b^As reported by young adult children. ***p < .001.

As Figure 1 shows, for authoritative parenting (*n* = 353), there was no direct effect of parents’ generativity on children’s attitudinal similarity to their parents. Furthermore, parents’ generativity had a simple indirect effect on children’s attitudinal similarity to their parents neither through parents’ reports on authoritative parenting, nor through children’s reports on authoritative parenting (see Table 2). The serial mediation of parents’ generativity through parents’ and children’s reports on authoritative parenting, however, yielded an indirect effect on attitudinal similarity, for which the confidence interval was entirely above zero. The direct and indirect effects summed up to a total effect of .153, *SE* = .053, *p* = .004, *R^2^* = .33.

**Table 2:** Completely Standardized Indirect Effects for the Relation Between Parents’ Generativity and Children’s Parent-Child Attitudinal Similarity per Parenting Style

	Simple indirect effects		Serial indirect effects
Parenting Style	generativity → parents’ reports on parenting style → attitudinal similarity	generativity → children’s reports on parenting style → attitudinal similarity	generativity → parents’ reports on parenting style → children’s reports on parenting style → attitudinal similarity
Authoritative parenting	.005 (.019) [–.032, .042]	.001 (.033) [–.061, .068]	.063 (.015) [.037, .095]
Authoritarian parenting	.001 (.006) [–.013, .015]	.001 (.010) [–.018, .023]	.001 (.006) [–.012, .012]
Permissive parenting	.002 (.005) [–.007, .013]	.008 (.013) [–.016, .035]	–.003 (.005) [–.013, .006]

*Note*. The table gives standardized regression coefficients, standard errors in brackets, and 95% confidence intervals in squared brackets. Simple/serial indirect effects are considered statistically significant when the 95% confidence interval does not include 0.

For authoritarian parenting (*n* = 347 because of the missing data), none of the indirect effects yielded a bootstrap confidence interval that did not include zero (see Table 2). Together with a significant direct effect of .155, *SE* = .052, *p* = .003, these indirect effects summed up to a total effect of .157, *SE* = .053, *p* = .003. Specifically, standardized regression coefficients were –.006, *p* = .914, for the path from parents’ generativity to parents’ reports on authoritarian parenting, .485, *p* < .001, for the path from parents’ to children’s reports on authoritarian parenting, and –.181, *p* = .002, for the path from children’s reports on authoritarian parenting on attitudinal similarity, respectively.

For permissive parenting (*n* = 347 because of the missing data), again, the significant total effect = .142, *p* = .008, was due to a significant direct effect = .135, *p* = .01. In addition, all of the indirect effects yielded a bootstrap confidence interval that included zero (see Table 2). In detail, standardized regression coefficients were –.042, *p* = .436, for the path from parents’ generativity to parents’ reports on permissive parenting, .301, *p* < .001, for the path from parents’ to children’s reports on permissive parenting, and .225, *p* < .001, for the path from children’s reports on permissive parenting on attitudinal similarity, respectively.

All analyses were rerun with parents’ age, gender, and educational status as well as children’s age, gender, and number of siblings as covariates. Results as described above were not affected by the inclusion of these covariates: Whereas the serial mediation was confirmed for authoritative parenting, the confidence intervals for all other indirect effect included zero. More detailed information on these additional analyses is presented in the Supplementary Materials, along with the data the present paper is based upon.

## Discussion

The present study was designed to replicate the findings by Peterson et al. (1997), which suggested a serial indirect effect of parents’ generativity on their young adult children’s evaluations of how similar their values are to those of their parents through the extent of authoritative parenting as reported by parents and children, with more power than the original study. Indeed, the serial mediation was confirmed. In other words, the more generative parents were, the more authoritative parenting they reported (cf. Pratt et al., 2001). Parents’ reports on authoritative parenting were associated with their children’s perception of authoritative parenting (cf. Lehmann et al., 2021). Finally, the more authoritative parenting children reported, the more similar they judged their attitudes to be to their parents’. The present study corroborates the mechanism proposed by Peterson et al. (1997) in that it fails to find indirect effects for authoritarian and permissive parenting. Hence, the serial mediation effect seems to be specific for authoritative parenting.

This result lends further evidence to the notion that adults’ generativity has some consequences for the recipient of the generative effort (Busch & Kranz, 2022; Jones & McAdams, 2013; Kessler & Staudinger, 2007). For example, research has shown that intergenerational contact programs have an effect on the generative individual (Gruenewald et al., 2016) as well as on the recipient of generative efforts (Thomas et al., 2022). Moreover, the results suggest one mechanism that might bring such generativity effects on the recipient about: At least in the context of parent–child dyads, authoritative parenting seems to be the link between generativity and its outcome. Thus, programs that aim at fostering parental skills (e.g., Joussemet et al., 2014) might help parents steer their generative concern towards the desired outcome of transmitting one’s attitudes and values.

It would be interesting to see how the proposed mechanism between adults’ generativity and recipients’ outcome translates to other generative contexts. For example, like parenting, mentor-mentee relationships also provide the generative adult with an on-going opportunity to pass on experiences and attitudes (Busch & Kranz, 2022; Jones & McAdams, 2013). Thus, as with parents, mentors who combine emotional support with standards and monitoring might be particularly successful in affecting their mentees’ attitudes. But what about short-lived interactions between a generative individual and the recipient of their generative efforts? Here, other mechanisms might be at work (such as, e.g., model learning, cf. Kessler & Staudinger, 2007), as there is no continuous opportunity for generativity.

To note, the present study is based on cross-sectional data and, thus, causality cannot be inferred. Naturally, a longitudinal verification of the present results would be desirable. Unfortunately, the present sample is restricted to university students (mostly majoring in the Humanities). Thus, the present results can hardly be generalized to young adults in general, because those in vocational training or apprenticeships are not represented in the present sample. Given that Peterson et al. (1997) also used a sample limited to university students, however, this limitation also has the advantage of increasing comparability with the original findings replicated here.

Furthermore, a more gender-balanced sample would make it possible to test if there are any differences between same-sex parent–child dyads and dyads of opposite sex. With a relatively small number of opposite-sex dyads, such analyses would not yield enough power to be meaningfully interpretable in the present sample. Of course, sampling both parents of each child would also further scientific knowledge on the circumstances under which adults’ generativity is successful in bringing parent-child attitudinal similarity about.

With respect to the operationalization of variables, the parent version of the Parental Authority Questionnaire was somewhat problematic in that two items did not load on the authoritative scale as expected. Although this has not affected the results of the serial mediation analysis, it seems advisable to test the results reported here again, but with a measure that has equivalent factor structures in its parent and child versions (for an example, see Lehmann et al., 2021). The use of the Parental Authority Questionnaire in the present study, however, is due to the present study’s attempt at directly replicating Peterson et al. (1997).

A further limitation of the present study is that children themselves rated their attitudinal similarity with their parent. Thus, in future studies, children and parents could be asked to report on their actual attitudes towards moral, social, cultural, and political issues. A confirmation of the present results for actual parent-child similarity would certainly strengthen the argument that adults’ generativity has an impact on the recipient. Again, it would also be highly informative to examine under which circumstances the generativity recipients react more positively to or elicit more generative efforts from their parents or other seniors. The present paper focusses on a unidirectional relationship between parental generativity and children’s perception of attitudinal similarity. However, as parenting styles and child characteristics are associated in a bidirectional way (e.g., Pinquart & Gerke, 2019), this bi-directionality ought to be represented in future generativity research as well.

Hopefully, the present study helps bring such open but vital questions for generativity research back to attention. As generativity is all about passing something on to future generations, generativity research should not only focus on the generative actor, but also investigate what the outcomes of generative efforts are. Supporting the findings by Peterson et al. (1997) with more power, the present study suggests that adults’ generative efforts can indeed influence their recipients’ attitudes.

## Supplementary Materials

For this article, the data that the study is based on as well as additional analyses results are available, see Busch (2022).



BuschH.
 (2022). Supplementary materials to "The association between parental generativity and parent-child attitude-similarity through parent- and child-reported authoritative parenting: A replication"
[Study data, additional analyses]. PsychOpen. https://osf.io/wjdnv
10.5964/ejop.8375PMC1093614038487318

## Data Availability

Data from the main and supplemental analyses are freely available, see Busch (2022).

## References

[sp1_r1] Busch H. (2022). Supplementary materials to "The association between parental generativity and parent-child attitude-similarity through parent- and child-reported authoritative parenting: A replication" [Study data, additional analyses]. PsychOpen. https://osf.io/wjdnv 10.5964/ejop.8375 PMC10936140 38487318

